# Effects of cardiopulmonary bypass on the accuracy of non-invasive hemoglobin measurement by pulse co-oximetry

**DOI:** 10.1186/2197-425X-3-S1-A745

**Published:** 2015-10-01

**Authors:** K Tokuda, K Yamaura, M Higashi, S Hoka

**Affiliations:** Intensive Care Unit, Kyushu University Hospital, Fukuoka, Japan; Department of Anesthesiology, Fukuoka University, Fukuoka, Japan; Department of Anesthesiology and Critical Care Medicine, Kyushu University Hospital, Fukuoka, Japan

## Introduction

A non-invasive continuous measurement of hemoglobin (Hb) by pulse co-oximetry (SpHb) using Radical-7^®^ (Masimo Corporation, CA, USA) is a newly developed method of measuring total Hb concentration. Previously, the accuracy of SpHb in clinical settings was reported (1). However, it has been also reported that SpHb has poor correlation with arterial Hb after cardiac surgery (2). Here we examined the accuracy of SpHb measured by New Radical-7^®^, which has new software version for measurement of total Hb, in cardiac surgery patients using cardiopulmonary bypass (CPB).

## Methods

Forty-six patients undergoing cardiac surgery using hypothermic CPB were included in this prospective study. Along with routine monitors, SpHb and direct radial arterial blood pressure were monitored. Arterial Hb by co-oximeter (Radiometer ABL 850; Radiometer, Copenhagen, Denmark) and SpHb with a new sensor (Revision K) for SpHb were simultaneously obtained and analyzed as paired data. SpHb was adjusted by “*in vivo* calibration” at start of operation, after CPB, and at transfer into the ICU. The data were shown as mean ± SD. Coefficient and Bland-Altmann analysis were used for analysis. Comparison of the mean differences was analyzed by Student's t-test with Bonferroni correction. *P* value < 0.05 was considered statistically significant.

## Results

A total of 386 time-matched SpHb and arterial Hb (before CPB in 67 pairs, after CPB in 81 pairs, and at transfer into the ICU in 238 pairs) were analyzed. SpHb ranged from 6.9 to 16.6 g/dl. The correlation coefficient was 0.87 before CPB, 0.61 after CPB and 0.72 in the ICU. The mean difference (SpHb - arterial Hb: bias) was 0.34 g/dl before CPB, -0.12 g/dl after CPB, and -0.19 g/dl in the ICU with 1SD for the bias 1.03 g/dl, 0.86 g/dl, and 1.11 g/dl, respectively. The correlation and mean difference between SpHb and arterial Hb showed good correlation before CPB when perfusion index was over 1.4. However, the correlation and mean difference between SpHb and arterial Hb after CPB and in the ICU did not show significant differences whether or not perfusion index was over 1.4.

## Conclusions

Our study showed that SpHb measured by New Radical-7^®^ deviated from arterial Hb measured by Radiometer ABL850^®^ after CPB; the difference did not depend on peripheral perfusion evaluated with perfusion index. These results suggest that some factors except for peripheral perfusion may decrease the accuracy of SpHb after CPB. Although non-invasive continuous Hb measurement by pulse co-oximetry is clinically useful in operating theater (3) and intensive care unit (4), actual Hb measurement of blood samples may remain to be needed after cardiac surgery using CPB.
